# A Feast of Reason

**Published:** 1891-12

**Authors:** 


### EDITORIAL DEPARTMENT.

F. E. DANIEL, M. D., EDITOR AND PUBLISHER.

THIS JOURNAL, by unanimous vote of the members, was made the Official Organ of the Austin District Medical Society.

COLLABORATORS:

*Dr. R. M. Swearingen, Austin.*
*Prof. B. E. Hadra, M. D., Galveston.*
*Prof. Geo. Cupples, M. D., San Antonio.*
*Dr. T. C. Osborn, Cleburne, Texas.*
*Dr E. J. Doering, Chicago.*
*Dr. E. J. Beall, Fort Worth.*
*Dr Odo Betz, Germany.*
*Prof. W. B. Rogers, M. D., Memphis.*
*Dr. J. W. McLaughlin, Austin.*
*Dr. T. J. Tyner, Austin.*
*Prof. J. F. Y. Paine, M. D., Galveston.*
*Dr. R. H. L. Bibb, Mexico.*
*Dr. T. J. Bennett. Austin.*
*Dr. Bat Smith, Wharton.*
*Dr. E. Meierhof, New York.*
*L. H. Luce, M. D., West Tisbury, Mass.*

### A FEAST OF REASON.

### AUSTIN DISTRICT MEDICAL SOCIETY—SEVENTEENTH QUARTERLY SESSION.

This flourishing and progressive organization held its seventeenth quarterly (fourth annual) meeting at Medical Hall, in Austin, on the 17th instant (December), with a very full attendance. Invitation had been sent to the Central Texas District Medical Association (Waco) to meet in joint session with the A. D., and there was a pretty full representation present from that Society.

Among those present were the venerable and witty Dr. J. H. Sears, of Waco; Dr. W. R. Blailock, of McGregor; Dr. J. H. Frazier, of Morgan; Dr. Foscue, of Waco; Dr. Gilsen, of Calvert; Drs. Reed and Pope, of Cameron, and many others whom the reporter does not now recall. Professors West and Thompson, of the Medical Department, University of Texas, Galveston, were present also, by invitation, and contributed much to the interest of the meeting. They brought pathological specimens, illustrating the lesions of certain cases of typhoid fever in which the diagnosis had been difficult, and also the heart of a patient who had died of organic disease of that viscus, under peculiar circumstances; also the larynx and trachea in a case of cut throat, in which the cut had been longitudinal instead of transverse. The remarks of the professors on these specimens were interesting and instructive in the highest degree, the discussion on typhoid fever taking a very wide range. Dr. Sear's remarks were both instructive and amusing. The doctor believes in quinine in typhoid fever, and practices what he preaches. He had just recovered from a long spell of fever, during which, he says, he took an ounce of quinine a week for several weeks—sixty to a hundred grains a day. He does not believe, however, he said, in over doing it;—the drug should not be abused,—but in moderate doses (?)—say ten, twenty or thirty grains every few hours, is about right in cases like his. The general sentiment of the meeting, however, appeared to be against quinine in typhoid fever. Until the diagnosis is clear, it is well to give a few doses of quinine in the earlier stage, before the disease has fully declared itself. The opinion appeared to prevail that after the first few days quinine not only does no good, but may, and probably does, do harm, bringing on hemorrhage or hæmaturia. Dr. Sears described the condition in which he found his left leg, upon convalescing, swollen and painful to the hip; he said it resembled *phlegmasia dolens*, and at first he thought he had turned to a woman. He asked for a diagnosis. Dr. Wooten believed it was due to *vaso-motor* paralysis, caused by the quinine. Dr. West and others held that it was thrombosis.

SEVERAL PAPERS

on typhlitis, and obstruction of the bowels, were read, and discussed at great length. One, by Dr. Parker, is published herewith. In the January number, we hope to give other papers, and a part of the discussion.

Dr. Weller read a paper on Syphilis, to open the subject, "Can syphilis be transmitted directly from the father to the child," he taking the affirmative. Dr. Richmond opened the discussion on the negative side, and for an hour the firing was very active all along the line. It was a drawn battle, however, and the subject is still *sub judice.*

THE ANNUAL ELECTION OF OFFCERS

for the Austin District Medical Society, resulted as follows:

President, Dr. G. W. Christian, Burnet.

First Vice-President, Dr. A. Garwood, New Braunfels.

Second Vice-President, Dr. W. T. Richmond, Manor.

Secretary and Treasurer, Dr. T. J. Bennett, Austin.

DR. J. W. M'LAUGHLIN'S

address as retiring president was upon "Contagion and Immunity," a subject which he handled with consummate skill and ability. It is a favorite subject with the doctor; one upon which he has thought and written much. He has a theory of his own, and accounts for immunity upon a new and original theory. In his address he demolishes Pasteur's theory, and the theory of "retention," and supports his own—the "doctrine of interference," with strong arguments. The address will be published. It was not discussed by any one but Dr. Sears, whose views on the subject of "germs" is well known. He says there are no "pathogenic bacteria;" the only function of any of the microbes is to remove or convert dead matter—they are scavengers.

